# Cannabidiol for Treatment of Childhood Epilepsy–A Cross-Sectional Survey

**DOI:** 10.3389/fneur.2018.00731

**Published:** 2018-09-07

**Authors:** Kerstin A. Klotz, Andreas Schulze-Bonhage, Victoria San Antonio-Arce, Julia Jacobs

**Affiliations:** ^1^Freiburg Epilepsy Center, Medical Center–University of Freiburg, Faculty of Medicine, University of Freiburg, Freiburg, Germany; ^2^Epilepsy Unit, Hospital Universitari Sant Joan de Déu, Barcelona, Spain

**Keywords:** cannabidiol, cannabinoids, CBD, children, EEG, electroencephalogram, epilepsy, seizure

## Abstract

**Background:** The interest in cannabidiol (CBD) for treatment of epilepsy has been increasing over the last years. However, practitioner's attitudes concerning the use of CBD for epilepsy treatment appears to be divided and data about its clinical use in daily practice are not available.

**Objective:** To improve the knowledge about the current use of CBD amongst European practitioners treating children and adolescents for epilepsy.

**Methods:** Cross-sectional survey using an open-access online questionnaire for physicians treating children or adolescents for epilepsy within eight European countries from December 2017 to March 2018.

**Results:** One-hundred fifty-five physicians participated in the survey. CBD is increasingly used by 45% (69/155) of participants, treating a mean (range) number of 3 (1–35) with CBD. Only 48% of the participants prescribing CBD are exclusively using purified CBD to treat children and adolescents with epilepsy, the remainder also applies preparations containing delta9-tetrahydrocannabinol (THC). Reported daily CBD doses range from < 10 to 50 mg/kg body weight. Management of CBD therapy in regard of monitoring side effects and adjusting concomitant therapy differs widely amongst participants. Their primary objective for commencing CBD is improving patient's quality of life. Participants frequently receive inquiries about CBD treatment but only 40% may actively suggest CBD as a treatment option. Of the 85 participants currently not using CBD for epilepsy treatment, 70% would consider using CBD if available in their country of practice or given the opportunity to become familiar with this treatment option.

**Conclusions:** CBD is increasingly used by participating physicians but individual experience remains limited. There are very diverse opinions about the use of CBD to treat epilepsy in children and adolescents and widely differing views on how to manage the CBD treatment.

## Introduction

Cannabidiol (CBD), an active cannabinoid without psychotropic effects and abuse liability, has recently gained interest as a treatment option for intractable epilepsy (1). CBD shows antiepileptic efficacy in acute and chronic seizure models in rodents but the precise mechanisms of action remain unclear (2–4). The clinical evidence to support the use of CBD is limited to retrospective case series (5–7), open label studies (8, 9) and two randomized controlled trials in children suffering from Dravet and Lennox-Gastaut syndrome (10, 11). Recent changes in the legal status of medical cannabis within several European countries have made CBD more accessible to health care professionals (12). However, practitioner's attitudes concerning the use of medical cannabis in general and CBD for treatment of epilepsy in particular appears to be divided (13, 14) and data about its clinical use in daily practice are not available.

The aim of this cross-sectional survey was to improve the knowledge about the current use of CBD amongst European practitioners treating children and adolescents for epilepsy. We surveyed practitioner's attitudes concerning the use of CBD, practical aspects of the drug treatment, i.e., dosing, management of side effects and of concurrent medication, and the practitioner's aim of initiating a CBD treatment. Furthermore, perceived indications as well as limitations for the use of CBD and legal issues for prescribing cannabinoids for medical purpose and reimbursement of treatment costs were assessed.

## Materials and methods

We developed a structured and stratified questionnaire using an online survey tool (SurveyMonkey, Portland, OR, USA) and invited physicians that are treating children and adolescents for epilepsy within eight different European countries to participate. Potential participants were identified via the regional subchapters of the International League Against Epilepsy and the respective national epilepsy organization homepages. We addressed potential participants either by contacting them directly or by using mailing lists.

The survey started with questions about the practitioner's professional qualification, their experience in treating pediatric epilepsy patients and their previous experience in using CBD for epilepsy in childhood.

From participants using CBD for childhood epilepsy, information about the following topics were requested: year of first use and total number of patients treated with CBD, treatment goals, dosing, monitoring and management of concurrent medication and of side effects of CBD as well as indications, limitations and contraindications for its use. Furthermore, interaction with patients and caregivers concerning the initiation of CBD, legislative regulation within the country of the survey participant, reimbursement of treatment costs and the use of cannabinoids other than CBD for epilepsy in childhood were inquired.

For physicians that were not treating patients with CBD, the survey proceeded with questions about the reasons for abstaining from its use and questions concerning the personal attitude toward CBD for epilepsy treatment in general. Finally, the therapeutic use of other cannabinoids, and the potential use of CBD, given any potential obstacles could be resolved, was inquired. The questionnaire is available as Supplementary Material.

Descriptive statistical analysis was performed with GraphPad Prism (V. 5.02, GraphPad Software, San Diego, CA, USA). Categorical variables are presented in absolute numbers and percentages and quantitative data as mean and standard deviation or median and interquartile range or range where applicable. Denominators are based on the number of answers for any given individual question.

## Results

One-hundred fifty-five physicians treating children and adolescents with epilepsy from eight different European countries participated in the survey (Table 1). Participants are qualified as board certified pediatric neurologists (110/155) neurologists (*n* = 36) or general pediatricians (*n* = 9). Sixty eight percent of all participants (106/155) completed a formal curricular training in epileptology. Sixty seven percent of all participants are affiliated with a neuropediatric or neurologic department of a medical facility (104/155), 25% work in a specialized epilepsy center (38/155), 3.5% in a general pediatric hospital (6/155), and 4.5% are engaged in private practice (7/155).

**Table 1:** Numbers of participants per country.

**Country**	**Number of participants**
Germany	62
Spain	41
Austria	18
Switzerland	13
Netherlands	8
Belgium	5
France	4
Italy	4
Total	155

**Figure 1:**
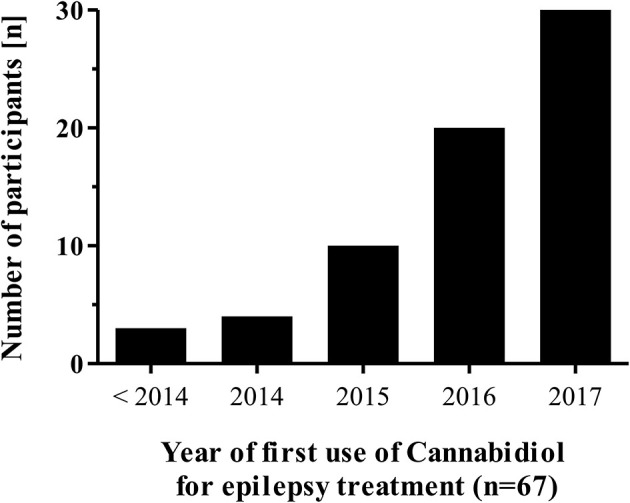
First year of Cannabidiol use for epilepsy treatment by individual participants (*n* = 67).

Overall, 45% (69/155) of respondents report a current or previous use of CBD for treating epilepsy in childhood. The participants' first use of CBD for this indication was in 2000 but increased only recently (Figure 1). CBD is used not only by epileptologists from specialized epilepsy centers (19/38), but also by those participants working in a neuropediatric/neurologic department (46/104) or by those working in either a private practice (2/7) or a general pediatric department (3/6).

**Figure 2:**
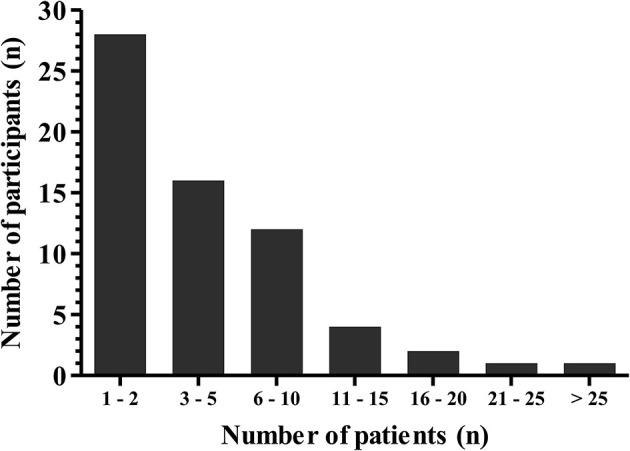
Number of patients per individual participant (*n* = 64) that were treated with Cannabidiol for epilepsy from 2004 until 2017.

**Figure 3:**
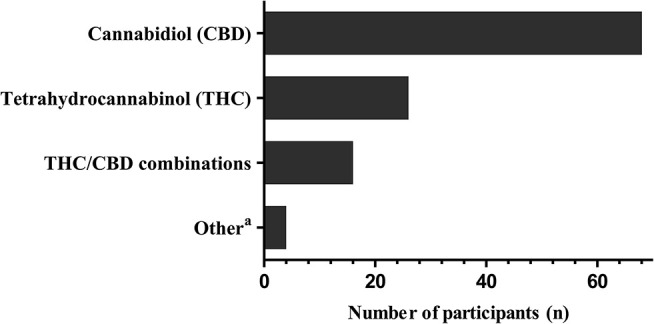
Cannabinoids used by participants for treatment of childhood epilepsy. Multiple answers were possible. ^a^Other: cannabidivarin (*n* = 1), homeopathic cannabis preparations (*n* = 2), vaporized medical cannabis (*n* = 1).

The median (range) number of CBD treated patients per CBD prescribing physician up to date is 3 (1–35) (Figure 2). Most participants (64%) initiate a CBD treatment based on an individual case by case decision, 21% of participants are using CBD almost exclusively within clinical trials and only 14% of participants follow a standardized departmental CBD treatment protocol. According to our survey, approximately 81% of the total number of patients (*n* = 356) of the CBD prescribing participants were treated outside of clinical trials. Ninety percent of participants are applying purified CBD preparations that are available by prescription only, in this instance the preparation is either dispensed by an institutional pharmacy (73% of cases) or manufactured by a licensed pharmaceutical company. However, 16% of the practitioners are using over the counter preparations to treat epilepsy with CBD. Only 48% of the participants are exclusively using purified CBD to treat children and adolescents with epilepsy, the remainder also applies delta9-tetrahydrocannabinol (THC) or TCH-CBD combination preparations (Figure 3).

For most participants prescribing CBD (*n* = 49) calculation of the daily CBD dose is based on body weight without an upper maximum dose, dose range was < 10 to 50 mg/kg body weight per day. However, whereas 73% of participants determined 10–25 mg/kg body weight to be the final CBD target dose, 24% aimed at a final target dose below 10 mg/kg and only two participants prescribed doses of above 25 mg/kg body weight per day. Fourteen participants refer to a maximum permissible CBD dose per day. However, nine of those participants could not specify this upper permissible dose range and the median (range) maximum permissible dose for the remainder (*n* = 5) was 600 (300–1,500) mg CBD per day regardless of the patient's body weight. CBD is usually given in two to three single doses per day and dosage is gradually increased by all respondents.

During CBD treatment, liver function tests are routinely performed by 72% of the participants, by 15% only in case of clinical symptoms or concomitant valproate treatment and 13% are not performing any liver function tests at all. CBD serum levels are rarely measured to adjust the treatment dose (*n* = 5). Management strategies of concomitant antiepileptic medication are given in Table 2.

**Table 2:** Management of preexisting anticonvulsive medication when commencing Cannabidiol.

	**Maintain unchanged *n* (%)**	**Reduce dose *n* (%)**	**Increase dose *n* (%)**	**Discontinue *n* (%)**	**Number of replies *n***
Clobazam	15 (33)	25 (54)	1 (2)	5 (11)	46
Phenobarbital	22 (58)	12 (31)	3 (8)	1 (3)	38
Phenytoin	24 (65)	9 (25)	2 (5)	2 (5)	37
Valproate	31 (67)	9 (20)	1 (2)	5 (11)	46
Carbamazepine	31 (80)	5 (13)	2 (5)	1 (2)	39
Topiramate	35 (83)	6 (14)	1 (3)	0 (0)	42
Zonisamide	34 (83)	6 (15)	1 (2)	0 (0)	41

**Figure 4:**
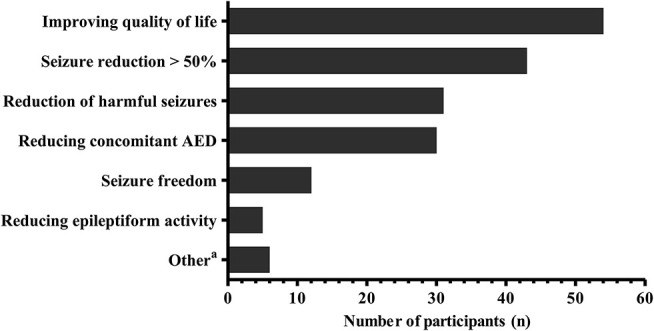
Treatment goals when commencing Cannabidiol for epilepsy according to the participants (*n* = 67). Multiple answers were possible. ^a^Other treatment goals include: seizure reduction < 50% (*n* = 2), spasticity (*n* = 4), pain (*n* = 2), anorexia (*n* = 1), sleep disturbance (*n* = 1). AED, antiepileptic drugs.

The vast majority of practitioners using CBD for epilepsy treatment (*n* = 50) would use CBD only in patients with proven pharmacoresistancy. The spectrum of treatment goals when initiating CBD is given in Figure 4. Remarkably, the primary objective appears to be improving the patient's quality of life. If treatment goals are not achieved, participants would discontinue the CBD treatment after a median (IQR) of 12 (12–20) weeks.

Perceived contraindications for commencing a CBD treatment in case of comorbidities are given in Table 3. Whereas some physicians would treat patients of any age with CBD (*n* = 6), most would not treat neonates (*n* = 44) or infants (*n* = 37). Furthermore, 22% of responders would treat all kinds of epilepsy etiologies with CBD. However, most responders would not use CBD for treating patients with genetic epilepsies other than Dravet syndrome, structural epilepsy (with the exemption of Lennox-Gastaut syndrome) or for less retractable epilepsies.

**Table 3:** Perceived contraindications for the use of Cannabidiol for treatment of epilepsy.

**Do you consider one of the following conditions as contraindication for the use of Cannabidiol?**			
***n*** **(%)**	**No contraindication**	**Relative contraindication**	**Absolute contraindication**
Failure to thrive	31 (61)	18 (35)	2 (4)
Elevated liver enzymes	11 (21)	37 (70)	5 (9)
Psychiatric comorbidities	22 (41)	27 (51)	4 (8)
History of or current substance abuse	17 (32)	16 (30)	20 (38)

A minority of participants regard the unconditional use of CBD for epilepsy treatment to be backed by the current body of evidence (*n* = 3). Fifty-six percent of the participants with previous CBD expertise acknowledge that data are limited but still may be sufficient to justify the use of CBD. The remainder (*n* = 30) may utilize CBD, either as a last resort (*n* = 16) or because they would rather commence a supervised CBD treatment before patients started self-medication (*n* = 14), even though data are insufficient for recommending a CBD treatment *per se*.

Ten percent of all survey participants receive enquiries concerning CBD treatment from parents and patients at least weekly, 28% at least monthly, 38% several times per year and 24% receive enquiries less frequently than that. Most participants discuss CBD as a treatment option only if requested by patients or their parents but 40% may actively suggest CBD as a treatment option.

CBD was never used to treat epilepsy by 55% of the participants (85/155). The lack of convincing evidence to support the use of CBD, the lack of personal experience and the restricted availability for legal reasons in some countries was cited as the main reason therefore (Supplementary Table 1). However, 70% of those practitioners would consider using CBD if available or permitted in their country of practice or given the opportunity to become familiar with this treatment option. Of those physicians not using CBD for epilepsy treatment, only 28% prescribed cannabinoids for other conditions, namely spasticity (*n* = 22), anorexia (*n* = 3), neurodegenerative disorders (*n* = 2) or movement disorders, hypersalivation, chronic pain syndromes, multiple sclerosis and self-regulation disorder (*n* = 1 each). In contrast, of those physicians having used CBD for epilepsy treatment, 47% prescribed cannabinoids for other conditions, namely spasticity (*n* = 27), multiple sclerosis (*n* = 4) or self-regulation disorder, anorexia, movement disorders and in palliative care scenarios (*n* = 3 each).

According to those participants who are using CBD, this cannabinoid is available for medical purposes in Spain, Germany, France, Austria, Switzerland and the Netherlands and not available in Belgium with missing data from Italy. Costs of CBD are covered by health care insurance providers within Spain, Germany, Switzerland and the Netherlands with missing data from France, Italy and Belgium. However, contradictory statements concerning availability and reimbursement are given by practitioners from three countries.

## Discussion

According to our survey, almost half of the participants have used or are using CBD for treatment of epilepsy in children and adolescents. The rising CBD prescription rate within the last years found in our survey corelates with a growing interest in medical cannabis in general and CBD in particular (1). However, the clinical evidence to support its use is scarce and the individual experience is mostly limited to a few patients per practitioner. Interestingly, even practitioners outside of specialized epilepsy centers und neuropediatric departments are using CBD in their patients. Given the lack of individual in depth experience in treating children and adolescents with CBD, a more centralized treatment approach may be suggested and treatment data should be prospectively collected.

CBD has been proven to be effective in acute and chronic seizure models in rodents (2, 3). However, sound clinical data to prove the efficacy of CBD, depending on the epilepsy type, are limited (15). Results of two randomized controlled trials on CBD as add-on anticonvulsant in patients with Dravet and Lennox-Gastaut syndrome have been published recently. In both trials CBD was efficient to reduce convulsive or drop seizures frequency respectively (10, 11).

Open label (8, 9), observational (16) and retrospective studies (5–7) indicate that other forms of intractable epilepsies may also respond to CBD. However, these observations are limited for various reasons (1). Consistently, structural or genetic epilepsies other than Dravet and Lennox-Gastaut syndrome were not considered to be treatable with CBD by most respondents of our survey. Unsurprisingly, given the limited data, survey participants would rather treat only patients with proven pharmacoresistancy, when other treatment options are exhausted or do not appear appropriate.

Randomized controlled trials primarily focus on seizure control as a main outcome criteria, implicating seizure control to be the primary treatment goal (10, 11). However, parental surveys and case reports suggest that from the patient's perspective, other treatment effects of CBD, e.g. improved sleep or behavior may be regarded as an equally beneficial treatment goal (5, 7, 9). Aforementioned aspects may impair the quality of life as much as the seizures themselves (17) and may be responsive to a CBD treatment (18). Interestingly therefore, in our participants opinion, improving the patient's quality of life was even more important than seizure reduction when initiating a CBD treatment. However, this may be in part due to the limited practitioner's expectation concerning seizure control in the respective patient population and we cannot provide information about decision making based on individual patient data. Nevertheless, increasing patient's quality of life appears to be a major motive for initiating a CBD treatment and should be included as outcome criteria in future CBD trials (19).

The two major neuroactive components of the cannabis plant out of more than hundred different cannabinoids are delta9-tetrahydrocannabinol (THC) and CBD (20). The latter cannabinoid is used by most participants. While the approval of the European Medicines Agency (EMA) is pending for a purified CBD pharmaceutical, purified CBD is currently only available as an individual pharmacy-dispensed preparation in some European countries or even prohibited in others. This may be the rationale to use over-the-counter preparations as reported by some participants, however, the reasons therefore are not elucidated by our survey. CBD enriched hemp oils are mixtures of cannabis extracts, presumably with a higher CBD content, and are freely available as dietary supplement. However, CBD concentrations in those preparations are not standardized. Quality controls revealed low CBD concentrations, potentially impacting anticonvulsive efficacy of the preparation, and revealed increased concentrations of THC in a large proportion of hemp oil samples (21). Therefore, when intending to initiate a CBD based treatment, there is currently no alternative available for individually dispended purified CBD preparations.

Preclinical data on THC prove anticonvulsant (22), as well as proconvulsive (23) and adverse effects on neurocognitive functioning (24). Clinical data show adverse structural and functional effects resulting from long term THC use (25, 26). Nevertheless, the use of THC containing preparations is still reported in our survey. Unlike CBD, the combination of THC and CBD (e.g. Sativex®) and dronabinol, a synthetic THC preparation (e.g. Marinol®), are EMA approved pharmaceuticals. This may facilitate its utilization and the approval of reimbursement from health care providers. Since clinical data concerning the safety and efficacy of THC or hemp oils for treatment of childhood epilepsy are not available, these preparations cannot be recommended.

A wide CBD dose range from < 10 mg/kg to 50 mg/kg body weight per day was reported by our survey participants. The dose that proved to be effective in two randomized controlled trials was 20 mg/kg, but open label trials suggest that individual patients required much higher doses up to 50 mg/kg for a response (8). However, dosing may depend on several factors, e.g. epileptic etiology, seizure types, age of the patient and comedications (7, 8, 27). Given the lack of data, an upper permissible dose cannot be defined and CBD doses up to 50 mg/kg were found to be safe (8, 28). Long term data of the use of CBD are equally limited (29). Therefore, there is no defined timeframe that qualifies as CBD treatment failure and this is reflected by the participant's divergent opinions about when to discontinue a CBD treatment.

The majority of participants in our survey would reduce clobazam when initiating a CBD treatment. While cytochrome P450 inhibiting CBD has shown relevant interactions with other anticonvulsant drugs in preclinical studies (30–32), the only clinically relevant interaction consists of increasing plasma concentrations of the active clobazam metabolite N-desmethyl-clobazam (8, 33–35). This interaction is claimed to be partially accountable for the anticonvulsant effect of CBD but also for increased sedation (8, 27, 33). Elevated aminotransferase levels during CBD treatment are reported to result almost exclusively from a concomitant valproate therapy (10, 33, 34). Nevertheless, liver enzymes are routinely measured by most participants regardless of comedication. Purified CBD preparations increase serum levels in a dose-dependent manner (33). However, pharmacokinetics of different CBD preparations or different routes of application and correlations between CBD serum level and anti-seizure effects or side effects have not been fully established (36, 37). Accordingly, almost none of the participants measure CBD serum levels.

There are several surveys of patients' or parents' opinions regarding CBD treatment for epilepsy (38–41), but only one that is including health care professionals. In that survey 52% of epileptologists and general neurologists would advise against using medical marijuana even in severe cases of epilepsy (14). In contrast, according to our survey the vast majority is using or would consider using CBD under certain circumstances, and only a minority would refrain from using it (*n* = 25) CBD. This may indicate a change of attitude concerning a cannabinoid-based treatment for epilepsy among practitioners treating children and adolescents for epilepsy.

According to our survey, most participants receive inquiries about CBD treatment on a regular basis. Interestingly, only about 40% would recommend CBD treatment actively whereas the majority discusses this treatment option only on request by patients or their parents. In our experience, families searching for alternative treatment options are burdened by a long history of seizures, side effects and complications. In an Australian nationwide survey on medicinal cannabis use for epilepsy, a significant proportion of children and adults with epilepsy were commencing cannabis-based products without medical supervision, even resulting in unsupervised reduction of concomitant antiepileptic treatment (41). Therefore, according to our survey some participants would rather commence a CBD treatment even if not convinced about its efficacy than having patients using it without medical supervision. However, decision to commence CBD treatment is mainly made on individual case-by-case basis and most patients of our participants are treated outside of clinical trials.

Interestingly, a substantial number of answers about their country specific availability and regulations concerning reimbursement of CBD were contradictory. This is in line with the results of an US survey about the use of medical cannabis in cancer patients. Only 5% of pediatric oncologists knew their state-specific regulations in this regard (42). This may indicate a further need for providing adequate information to health care providers given the striking differences between European countries in regulation, availability and covering of costs of medical cannabinoids in general and CBD in particular (12).

Given the widely differing practice concerning indications and limitations, choice of preparation, dosing and monitoring revealed by our survey, official guidelines for the use of CBD for epilepsy treatment appear to be advisable to harmonize and potentially improve its use.

There are several limitations concerning our survey. Since this was an open-access survey we cannot generate a response rate. We relied onto the participants to reply truthfully and thoroughly. Numbers and percentages of CBD prescribers may be overestimated by a participation bias, that may be indicated by a substantial variation of responses between countries. Furthermore, we were not able to relate the numbers of participants to the total number of physicians that are treating children and adolescents with CBD in participating countries. Therefore, we cannot to draw a representative picture for the extent of CBD use in participating countries. These aspects need to be considered when interpreting our findings.

Nevertheless, we presented a broad overview of certain aspects of CBD use by European experts of childhood epilepsy and highlighted several limitations for its use in clinical practice.

## Conclusions

CBD appears to be increasingly used by the participants of our survey, but the individual experience remains limited. There are diverse opinions about the use of CBD to treat epilepsy in children and adolescents and widely differing views on several aspects in managing the CBD treatment.

## Ethics statement

This study was approved by the Ethics Committee of the Albert-Ludwigs-University Freiburg, Germany (No. 68/18).

## Author contributions

KK conceptualized and designed the questionnaire, collected contact data, performed the survey, analyzed data and drafted the initial manuscript. VS contributed to data collection, and revised the manuscript. AS-B contributed to the design of the questionnaire and revised the manuscript. JJ contributed to the design of the manuscript, contributed to the collection of contact data and revised the manuscript. All authors gave final approval of the version to be published. All authors agree to be accountable for the content of the work.

### Conflict of interest statement

The authors declare that the research was conducted in the absence of any commercial or financial relationships that could be construed as a potential conflict of interest.
