# Supplementary material for: Cannabidiol for Treatment of Childhood Epilepsy–A Cross-Sectional Survey
Source: Front Neurol. 2018 Sep 7;9:731. doi: 10.3389/fneur.2018.00731 (PMC6143823; doi:10.3389/fneur.2018.00731)
Supplement: Supplementary file 1 [file Data_Sheet_1.PDF]

Supplementary Material

Questionnaire

Cannabidiol for Treatment of Childhood Epilepsy – a cross-sectional Survey

Kerstin Alexandra Klotz*, Andreas Schulze-Bonhage, Victoria San Antonio-Arce, Julia Jacobs

***Corresponding Author**: kerstin.alexandra.klotz@uniklinik-freiburg.de

This is the PDF-Version of a stratified and responsive digital online-Survey. Skip logic is not depicted.

* 1. Are you treating children or adolescents with epilepsy ?

- ◯ Yes and I am a paediatrician
- ◯ Yes and I am a neurologist
- ◯ No

Professional qualification

2. Are you a board certified Neuropaediatrician ?

- ◯ Yes
- ◯ No
- ◯ Other (please specify)

3. Have you completed a formal curricular training in Epileptology?

- ◯ Yes
- ◯ No
- ◯ Other (please specify)

4. I am affiliated with / I am working in a

- ◯ Private practice / doctor's office
- ◯ Neuropaediatric/Neurological Department
- ◯ Epilepsy Center
- ◯ other (please specify)

Previous experience with the use of Cannabidiol

* 5. Have you ever prescribed Cannabidiol (CBD) for treating epilepsy in children or adolescents?

- ◯ Yes
- ◯ No

Previous experience with the use of Cannabidiol

6. What were the treatment goals in your patients treated with Cannabidiol ? (Multiple answers possible)

- [ ] Seizure freedom
- [ ] Seizure reduction of ≥ 50%
- [ ] Reduction of potentially harmful seizures ("Grand mal seizures", drop seizures)
- [ ] Reduction of epileptiform activity in EEG
- [ ] Improving patients' quality of life
- [ ] Reducing dose or number of other antiepileptic drugs
- [ ] others (please specify)

7. How many patients have you treated with Cannabidiol to date ?

Number of patients treated with Cannabidiol:

8. In which year did you start treating patients with Cannabidiol ?

Year

Application of Cannabidiol

9. Which Cannabidiol preparations are you using ? (Multiple answers possible)

- [ ] Preparations manufactured by a pharmacy only available on prescription
- [ ] Preparations manufactured by a licensed pharmaceutical company only available on prescription
- [ ] Over the counter preparations (e.g. dietary supplements)
- [ ] other (please specify)

10. Have you ever used Cannabinoids other than Cannabidiol (CBD) to treat epilepsy in children or adolescents? (Multiple answers possible)

- [ ] No
- [ ] Tetrahydrocannabiol (THC, e.g. Dronabinol)
- [ ] Combination of Tetrahydrocannabiol + Cannabidiol
- [ ] Hemp (oil)
- [ ] Homeopathic cannabinoid preparations
- [ ] Vaporized medical cannabis
- [ ] Others (please specify)

Dosing of Cannabidiol

11. What is your final target dose when treating epilepsy with Cannabidiol (mg/kg bodyweight) ?

- ◯ < 10 mg/kg
- ◯ 10 - 25 mg/kg
- ◯ > 25 mg/kg
- ◯ other (please specify)

12. This is divided into how many single doses of Cannabidiol per day ?

- ◯ 1
- ◯ 2
- ◯ other (please specify)

13. Do you gradually increase the Cannabinidiol dose ?

- ◯ Yes, gradually increase
- ◯ No, start with final target dose
- ◯ Other (please specify)

14. In your opinion is there a maximum permissible Cannabidiol dose ?

- ◯ Yes
- ◯ No, dosage should be based on body weight
- ◯ others (please specify)

Dosing of Cannabidiol

15. The maximum permissible daily dose of Cannabidiol is

dose in milligrams (mg)

Monitoring & management of side effects

16. Do you perform liver function tests during Cannabidiol treatment ?

- ◯ No
- ◯ Routinely
- ◯ Only in case of clinical signs indicating adverse effects
- ◯ Only in case of a co-medication with Valproate
- ◯ Other (please specify)

17. Do you consider one of the following conditions to be contraindications for the use of Cannabidiol?

	No contraindication	Relative contraindication	Absolute contraindication
Failure to thrive	◯	◯	◯
Elevated liver enzymes	◯	◯	◯
Psychiatric comorbidities	◯	◯	◯
History of or current substance abuse	◯	◯	◯
Obesity	◯	◯	◯

18. How do you proceed with preexisting anticonvulsive medications ?

	Discontinue Drug	Maintain dose unchanged	Reduce dose	Increase dose
Valproate	◯	◯	◯	◯
Clobazam	◯	◯	◯	◯
Phenobarbital	◯	◯	◯	◯
Phenytoin	◯	◯	◯	◯
Carbamazepine	◯	◯	◯	◯
Topiramate	◯	◯	◯	◯
Zonisamide	◯	◯	◯	◯

other (please specify)

19. Do you measure serum levels of Cannabidiol to adjust treatment dose ?

- Yes
- No
- Other (please specify)

Indication for the use of Cannabidiol

20. Would you consider using Cannabidiol as

- ◯ First-line treatment ?
- ◯ Second-line treatment ?
- ◯ after proven pharmcoresistancy only (definition according to ILAE) ?
- ◯ other (please specify)

21. On what basis did you or would you decide to commence a Cannabidiol treatment ? (Multiple answers possible)

- ☐ As part of a standardized departmental treatment protocol (SOP)
- ☐ Individual decision
- ☐ Only in clinical trials
- ☐ others (please specify)

22. Is the use of Cannabidiol for seizure treatment justified by the current body of evidence on this topic (as of December 2017) ? (Multiple answers possible)

- ☐ Yes, without question
- ☐ Data are limited, but current evidence from clinical experience justifies its use
- ☐ Despite limited data, Cannabidiol may be used as a last resort only
- ☐ No, current evidence does not justify its use; but my patients may start self medication therefore I would rather commence a supervised Cannabidiol treatment
- ☐ Other (please specify)

Limitations and contraindications for the use of Cannabidiol

23. After how long should Cannabidiol be discontinued if treatment goals have not been achieved ?

weeks

months

24. In your opinion, which forms of epilepsy should **NOT** be treated with Cannabidiol? (Multiple answers possible)

- ☐ Dravet syndrome
- ☐ other genetic epilepsy
- ☐ Lennox-Gastaut syndrome (LGS)
- ☐ structural epilepsy without LGS
- ☐ other (please specify)

25. Which age group(s) should **NOT** be treated with Cannabidiol due to potential**safety** issues ? (Multiple answers possible)

- ☐ Neonates (day of life 0-28)
- ☐ Infants (day of life 28 - 1 year of age)
- ☐ Toddlers (1 - 5 years of age)
- ☐ School children (6 - 12 years of age)
- ☐ Adolescents (13 - 18 years of age)
- ☐ All of the above could be treated with Cannabidiol
- ☐ Other (please specify)

Interaction with patients / parents

26. How often do you receive enquiries concerning Cannabidiol treatment from parents / patients ?

- ◯ Very often (at least weekly)
- ◯ Often (at least monthly)
- ◯ Occasionally (several times yearly)
- ◯ Rarely (less then above)
- ◯ Never
- ◯ Other (please specify)

27. Do you actively suggest to parents / patients to start a treatment with Cannabidiol ?

- ◯ Yes, I may suggest treatment to parents / patients
- ◯ No, I only discuss this treatment option if parents / patients enquire about Cannabidiol
- ◯ Other (please specify)

Regulation and Reimbursement

28. What is the legal basis for the medical use of Cannabidiol in your country / jurisdiction ?

- ◯ Available for medical purposes
- ◯ Available for medical purposes after individual application and official approval of authorities
- ◯ Freely Available (without a prescription)
- ◯ Possession and use prohibited
- ◯ Other (please specify)

* 29. How is reimbursement for the costs of Cannabidiol treatment regulated ?

- ◯ Reimbursed by the health insurance
- ◯ Reimbursed by the health insurance after individual application
- ◯ No reimbursement provided (guardians/patient have to pay out-of-pocket)
- ◯ Not applicable
- ◯ Other (please specify)

30. How often do you receive enqueries concerning Cannabidiol treatment from parents / patients ?

- ◯ Very often (at least weekly)
- ◯ Often (at least monthly)
- ◯ Occasionally (several times yearly)
- ◯ Rarely
- ◯ Never
- ◯ Other (please specify)

* 31. What is your motivation not to use Cannabidiol to treat intractable epilespy in children or adolescents ? (Please provide the answer that applies most)

- ◯ Not available in my country / jurisdiction
- ◯ Cannabidiol treatment will be commenced by other colleagues within my team or department
- ◯ No medical need to use Cannabidiol in my patients so far
- ◯ No personal experience
- ◯ Not enough evidence to support the use of Cannabidiol
- ◯ I do not condone the use of cannabinoids for medical purposes
- ◯ Other (please specify)

* 32. If it was available within your country / jurisdiction would you consider using it ?

- Yes
- No, not enough evidence to support the use of Cannabidiol
- No, there is generally no indication for Cannabidiol when treating epilepsy
- No personal experience
- I dont condone the use of cannabinoids for medical purposes
- Other (please specify)

* 33. If you had the chance to become familiar with this drug, would you then consider using it?

- Yes
- No medical need to use Cannabidiol in my patients
- Not enough evidence to support the use of Cannabidiol
- I dont condone the use of cannabinoids for medical purposes

34. Could you provide us with the contact details of the respective colleague ?

Previous experience with Cannabinoids

35. Have you ever used Cannabinoids for other conditions? (Multiple answers possible)

- ☐ No
- ☐ Epilepsy
- ☐ Spasticity
- ☐ Anorexia
- ☐ Self-regulation disorder
- ☐ Multiple sclerosis
- ☐ Other (please specify)

Thank you very much for participating in our survey !

Please let us know if you have any questions concerning this survey:

kerstin.alexandra.klotz@uniklinik-freiburg.de
