# Supplementary material for: Cannabidiol for Treatment of Childhood Epilepsy–A Cross-Sectional Survey
Source: Front Neurol. 2018 Sep 7;9:731. doi: 10.3389/fneur.2018.00731 (PMC6143823; doi:10.3389/fneur.2018.00731)
Supplement: Supplementary file 2 [file Data_Sheet_2.PDF]

Supplementary Material

Cannabidiol for Treatment of Childhood Epilepsy – a cross-sectional Survey

Kerstin Alexandra Klotz*, Andreas Schulze-Bonhage, Victoria San Antonio-Arce, Julia Jacobs

***Corresponding Author**: kerstin.alexandra.klotz@uniklinik-freiburg.de

Supplementary table 1 | Participants' main reasons for not using Cannabidiol for epilepsy treatment

Answers	n (%)
Not enough evidence to support the use of Cannabidiol	25 (32)
Not available/permitted in my country of practice	23 (29)
No personal experience	22 (28)
No medical reason to use Cannabidiol in my patients	8 (10)
Uncertainty of reimbursement	1 (1)
